# Hepatitis C Virus in Pregnancy: Case Reports and Literature Review

**DOI:** 10.1155/S1064744995000731

**Published:** 1995

**Authors:** Gary M. Joffe

**Affiliations:** Department of Obstetrics and Gynecology Lovelace Medical Center 5400 Gibson, S.E. Albuquerque NM 87108 USA

## Abstract

*Background:* Hepatitis C virus (HCV) is now recognized as the cause of 90% of non-A, non-B (NANB) hepatitis. This virus is responsible for a large percentage of chronic persistent and chronic active hepatitis in the United States. Parenteral and sexual transmission are well described, so a significant population of pregnant patients is at risk. Vertical transmission of the virus to the fetus is dependent upon the level of maternal viremia.

*Case:* The cases described in the following report demonstrate that fulminant disease may present in pregnancy. They also demonstrate the cofactors promoting the severity of illness, methods of diagnosis, potential treatment, and outcome of the infection.

*Conclusion:* HCV may be encountered in pregnancy. Although most acute-phase illness will be self limiting, some patients will manifest liver failure during gestation. Because vertical transmission to the fetus is possible, the pediatrician should be informed of the maternal disease. Chronic hepatitis is almost the rule rather than the exception, so patients require close postpartum follow-up. Interferon, which may alter the course of the chronic disease, has been used on rare occasions in pregnancy.


Infectious Diseases in Obstetrics and Gynecology 3:248–251 (1995)
© 1996 Wiley-Liss, Inc.

### Hepatitis C Virus in Pregnancy: Case Reports and Literature Review

Gary M. Joffe

*Department of Obstetrics and Gynecology, Lovelace Medical Center, Albuquerque, NM*

ABSTRACT

***Background:*** Hepatitis C virus (HCV) is now recognized as the cause of 90% of non-A, non-B (NANB) hepatitis. This virus is responsible for a large percentage of chronic persistent and chronic active hepatitis in the United States. Parenteral and sexual transmission are well described, so a significant population of pregnant patients is at risk. Vertical transmission of the virus to the fetus is dependent upon the level of maternal viremia.

***Case:*** The cases described in the following report demonstrate that fulminant disease may present in pregnancy. They also demonstrate the cofactors promoting the severity of illness, methods of diagnosis, potential treatment, and outcome of the infection.

***Conclusion:*** HCV may be encountered in pregnancy. Although most acute-phase illness will be self limiting, some patients will manifest liver failure during gestation. Because vertical transmission to the fetus is possible, the pediatrician should be informed of the maternal disease. Chronic hepatitis is almost the rule rather than the exception, so patients require close postpartum follow-up. Interferon, which may alter the course of the chronic disease, has been used on rare occasions in pregnancy. © 1996 Wiley-Liss, Inc.

KEY WORDS

4-RIBA, vertical transmission, blood transfusion

The hepatitis C virus (HCV) is now recognized as one of the leading causes of chronic liver disease and cirrhosis throughout the world. The identification of the viral genome has led to the awareness that, although almost all post-transfusion hepatitis cases are the result of this agent, a blood transfusion is not the major source of the disease. Two cases of HCV infection in pregnancy are presented along with a discussion of the pathogenesis, diagnosis, outcome, and treatment of this devastating chronic disease.

### CASE REPORTS

### Case 1

The patient was a 34-year-old $G_2P_1$ with an estimated gestational age at admission of 34 weeks. Eight days prior to her admission, she noted the onset of watery stool and mild epigastric pain. Other signs included dark urine and jaundice on the day prior to her admission. She denied any medication, alcohol, or toxin exposure or household contacts with hepatitis. Her physical examination at admission was significant for a blood pressure of 100/60 and a temperature of 36.2°C. Her sclera were icteric and her skin was deeply jaundiced. Her abdomen was nontender with no hepatomegaly. Her admission laboratory values included an AST of 1,195 (normal range 3–50 IU/l), ALT of 796 (normal range 9–52 IU/l), and total bilirubin of 5.9 (normal range 0.2–1.3 mg/dl). Additional laboratory values included a platelet count of 279,000 and an LDH of 775 (normal range 313–618 IU/l). The prothrombin time (PT) and partial thromboplastin time (PTT) were 10.3 and 29, respectively (normal range PT

Address correspondence/reprint requests to Dr. Gary M. Joffe, Department of Obstetrics and Gynecology, Lovelace Medical Center, 5400 Gibson, S.E., Albuquerque, NM 87108.

Received September 25, 1995
Accepted December 29, 1995

11.1–14.7 sec, PTT 20–33 sec). A right upper-quadrant ultrasound demonstrated normal gallbladder anatomy with no evidence of cholelithiasis. The admission nonstress test was reactive. The differential diagnosis included infectious hepatitis, severe preeclampsia, fatty liver of pregnancy, autoimmune hepatitis, and Wilson's disease.

Hepatitis A and B testing was negative, as was herpesvirus IgM. The serum ceruloplasmin, ANA, iron, and ferritin were normal. A hepatitis C antibody was ordered, but the results were not immediately expected. The 24-h urine protein was 324 mg, with a creatinine clearance of 143 ml/min. An amniocentesis was performed on the day of admission to assess the need for steroids to enhance the fetal lung maturity. The amniocentesis was performed prior to the deterioration in the maternal hepatic function. The values revealed a lecithin/sphingomyelin ratio of 2.2, with phosphatidyl glycerol present. The amniotic-fluid glucose was 26 mg/dl. An ultrasound demonstrated a singleton pregnancy in a breech presentation with an estimated fetal weight of 2,213 g.

On hospital day 4, the patient's PT rose to 11.2, with an International Normalized Ratio (INR) of 1.3. With the rising PT in the presence of documented fetal lung maturity, the patient was offered delivery by a primary cesarean with an intraoperative liver biopsy. A cesarean delivery was offered for the breech presentation. The neonate weighed 2,128 g, with Apgar scores of 8 and 9.

The patient's postoperative course was complicated by a slow resolution of her hepatic dysfunction. The liver biopsy demonstrated active hepatitis with necrosis but no fatty infiltration. The serum hepatitis C antibody by enzyme-linked immunosorbent assay (ELISA) was positive when the results returned 5 weeks post-delivery, having been sent to an outside reference laboratory with slow turnaround time. The liver biopsy also demonstrated HCV RNA by the polymerase chain reaction (PCR) method. Her husband was HCV negative by ELISA. One year after delivery, she demonstrated chronic active hepatitis, with the AST persisting at 115–120 and ALT at 160–165.

### Case 2

The patient was a 35-year-old $G_3P_{2002}$ who presented to the emergency room with severe epistaxis at 17.5 weeks estimated gestational age. After the bleeding was successfully controlled with nasal packing, a physical examination revealed a morbidly obese female whose spleen edge was 4–5 cm below the left costal margin. Her laboratory values were significant for normal hemoglobin and hematocrit, but a platelet count of 81,000. The PT was 16.4 with an INR of 1.6, and the PTT was 46. The liver enzymes included an AST of 92, ALT of 75, and total bilirubin of 0.9. An abdominal ultrasound revealed the spleen to measure 17.4 cm in superior-to-inferior dimension. Upon further questioning, the patient stated that she had a longstanding history of intravenous (IV)-drug abuse, but had not used drugs in this fashion for several years. However, she did state that she had episodes of "binge" drinking in which she consumed at least a quart of beer at a time. Her most recent episode had occurred 4 days prior to her admission. Severe portal hypertension was suspected based upon the above findings, with significant platelet sequestration in the maternal spleen. A workup for autoimmune liver disease, antiphospholipid antibody disease, hemochromatosis, and Wilson's disease was negative. The patient had a positive 4-recombinant immunoblot assay (4-RIBA) for HCV with antibodies detected against 4 antigens encoded by different parts of the HCV genome. Despite several more episodes of epistaxis, the only other complication during the remainder of her pregnancy was insulin-requiring gestational diabetes. At 38 weeks gestation, an induction of labor was offered. On the day of her admission, the laboratory values included a PT of 16.7, PTT of 46, and platelet count of 94,000. When the active phase of labor was achieved, the patient was given 2 units of fresh-frozen plasma. At the time of complete cervical dilation, she was given 6 units of platelets. Clotting factors were administered because of her increased risk for postpartum hemorrhage as well as bleeding from the suspected esophageal varices. After a passive second stage (minimal maternal pushing efforts), she had a spontaneous vaginal delivery of a viable 4,341-g female infant. There was no obstetric hemorrhage or rupture of the esophageal varices during delivery. The cord blood obtained for determination of HCV RNA status was negative by PCR.

### DISCUSSION

In 1989, the genome of HCV was cloned, and an assay was developed to detect the presence of antibodies directed against this agent.[1] Subsequently, with the use of radioimmunoassay (RIA), ELISA, and RIBA, it was demonstrated that approximately 88–90% of post-transfusion non-A, non-B (NANB) hepatitis is associated with hepatitis C infection.[2,3] The incidence of NANB, most of which is accounted for by HCV, has been reported as 7.1 per 100,000 cases.[4] This RNA virus has not yet been visualized. The incubation period is 7 weeks, and the mean time interval between transfusion and anti-HCV seroconversion is 16–22 weeks.[2,3] Transaminase levels range from 450 to 2,000 IU/l.[3] The mean length of time for an elevation of transaminases in patients with resolving hepatitis has been reported as 8.2 weeks.[3] The bilirubin levels are significantly elevated in over 75% of patients.[4] The mortality rate following the acute onset of the disease has been reported as 1.6%.[4] An estimated 40–60% of transfusion-associated HCV infections result in chronic disease, with 20–40% eventually developing cirrhosis.[5] The combination of chronic alcohol exposure and HCV infection results in significantly greater liver injury evident on histologic examination than the presence of alcohol exposure alone.[6]

Several factors have significantly decreased the incidence of transfusion-associated HCV infection. These include testing blood donors for levels of ALT as well as antibody to hepatitis B core antigen, which started in 1986. Screening for human immunodeficiency virus infection also contributed to the decline. Screening of potential blood donors along with a significant increase in IV-drug abuse in association with HCV infection between 1984 and 1989 has led to the conclusion that most cases of NANB hepatitis in the United States are not transfusion related.[4] Approximately 45–50% of NANB hepatitis are associated with IV-drug abuse. Blood transfusion accounts for as few as 2% of cases. Household contacts of infected individuals represent 10–15% of the patients with HCV infections. The source of infection is unidentified in roughly 35% of cases; case-control studies have demonstrated a significant association with multiple sexual partners in the heterosexual population.[4]

Screening for HCV infection is usually carried out in two steps. The ELISA, a somewhat nonspecific assay for anti-HCV antibody, is the first step in the screening process. Over half of those found to be positive on the ELISA screen will prove to have false-positive results when the more specific 4-RIBA is carried out. This assay requires that at least 2 of 4 HCV antigens be present, as demonstrated by antibody reactivity. The 4-RIBA has demonstrated a strong correlation with the PCR determination of HCV sequences in the sera of patients with post-transfusion NANB hepatitis.[7]

In 1989, it was demonstrated that many patients who developed fulminant infectious hepatitis were completely lacking in endogenous interferon production.[8] In an uncontrolled trial, these investigators suggested that treatment with human interferon alpha decreased the mortality associated with severe disease resulting in hepatic coma. This series included 3 pregnant patients. Two of these 3 patients survived.

Maternal-to-infant transmission of HCV is thought to occur less frequently than with HBV infection. However, the recent literature suggests a strong positive correlation between the level of maternal viremia at the time of delivery and the incidence of HCV infection in the infant. The degree of clinical disease, in other words, chronic active hepatitis vs. chronic persistent hepatitis, is also predictive of transmission to the offspring of infected mothers.[9] Although the number of mother-infant pairs studied is limited, the generally quoted risk for the vertical transmission of HCV is 5–10%. Presently, there are no tests available to detect the presence of HCV antigen in the serum. Therefore, it is not possible to determine level of infectivity as in the case of hepatitis B infection where hepatitis B e antigen can be assayed. The PCR for amplification of complementary nucleotide sequences to portions of the viral genome is still not available in most hospital laboratory settings. Furthermore, a single negative PCR examination does not eliminate the possibility of HCV infection, as titers of viral nucleic acids fluctuate widely throughout the course of the chronic disease. The role of breastfeeding in the transmission of HCV is incompletely defined, although transmission by this route has been described.[10]

Case 1 demonstrates that fulminant hepatitis secondary to HCV infection can occur in pregnancy. This patient was typical in that the source of her exposure could not be identified. Unfortunately, she became 1 of the 40–60% of patients developing chronic hepatic dysfunction. Case 2 demonstrates the severe hepatic destruction that can occur when a patient with persistent HCV replication adds ethanol intake to the equation. Indeed, a significant population of patients requiring liver transplantation in the United States present with both of these environmental exposures as etiologies for their disease. With the severity of her disease, this patient was fortunate in that she did not manifest vertical transmission of the HCV agent.

In conclusion, pregnancy-associated HCV infection may present as a mild, self-limited disease, chronic disease, or fulminant hepatitis. Most infections are not sequelae of blood transfusions but more often associated with IV-drug abuse. Although the HCV antigen cannot yet be demonstrated in serum, antibody testing by ELISA with confirmation by the 4-RIBA method is now readily available. Vertical transmission may be demonstrated by the presence of viral RNA sequences using PCR techniques.

Primary-care obstetrician-gynecologists can expect to manage patients with subclinical but persistent HCV infections, either during pregnancy or during routine gynecologic care. As such, these physicians must be aware that a high percentage of patients develop chronic hepatic dysfunction and that alcohol consumption significantly accelerates the disease process. In addition, patients should be aware that, at the present time, interferon is the mainstay of therapy in the presence of chronic active disease.

### REFERENCES

1. Kuo G, Choo QL, Alter HJ, et al: An assay for circulating antibodies to a major etiologic virus of human non-A, non-B hepatitis. Science 244:362–364, 1989.
2. Esteban JI, Gonzalez A, Hernandez JM, et al: Evaluation of antibodies to hepatitis C virus in a study of transfusion-associated hepatitis. N Engl J Med 323:1107–1112, 1990.
3. Alter HJ, Purcell RH, Shih JW, et al: Detection of antibody to hepatitis C virus in prospectively followed transfusion recipients with acute and chronic non-A, non-B hepatitis. N Engl J Med 321:1494–1500, 1989.
4. Alter MJ, Hadler SC, Judson FN, et al: Risk factors for acute non-A, non-B hepatitis in the United States and association with hepatitis C virus infection. JAMA 264:2231–2235, 1990.
5. Stein JH: Internal Medicine. CD-ROM Edition. St. Louis: C.V. Mosby, Part 4E, 1995.
6. Pares A, Barrera JM, Caballeria J, et al: Hepatitis C virus antibodies in chronic alcoholic patients: Association with severity of liver injury. Hepatology 6:1295–1299, 1990.
7. Van Der Poel CL, Cuypers HTM, Reesink HW, et al: Confirmation of hepatitis C virus infection by new four-antigen recombinant immunoblot assay. Lancet 337: 317–319, 1991.
8. Levin S, Leibowitz E, Torten J, Hahn T: Interferon treatment in acute progressive and fulminant hepatitis. Isr J Med Sci 25:364–372, 1989.
9. Ohto H, Terazawa S, Sasaki N, et al: Transmission of hepatitis C virus from mothers to infants. N Engl J Med 330:744–750, 1994.
10. Gurakan B, Oran O, Yigit S: Vertical transmission of hepatitis C virus (letter). N Engl J Med 331:339, 1994.
